# Experimental Study on Variation of Surface Roughness and Q Factors of Fused Silica Cylindrical Resonators with Different Grinding Speeds

**DOI:** 10.3390/mi12091052

**Published:** 2021-08-30

**Authors:** Libin Zeng, Yunfeng Tao, Yao Pan, Jianping Liu, Kaiyong Yang, Hui Luo

**Affiliations:** College of Advanced Interdisciplinary Studies, National University of Defense Technology, Changsha 410073, China; zenglibin19@nudt.edu.cn (L.Z.); taoyunfeng13@nudt.edu.cn (Y.T.); l_jianp@sina.com (J.L.); yky208@nudt.edu.cn (K.Y.); luohui.luo@163.com (H.L.)

**Keywords:** quality factor, resonator, subsurface damage, surface roughness

## Abstract

For the axisymmetric shell resonator gyroscopes, the quality factor (Q factor) of the resonator is one of the core parameters limiting their performances. Surface loss is one of the dominating losses, which is related to the subsurface damage (SSD) that is influenced by the grinding parameters. This paper experimentally studies the surface roughness and Q factor variation of six resonators ground by three different grinding speeds. The results suggest that the removal of the SSD cannot improve the Q factor continuously, and the variation of surface roughness is not the dominant reason to affect the Q factor. The measurement results indicate that an appropriate increase in the grinding speed can significantly improve the surface quality and Q factor. This study also demonstrates that a 20 million Q factor for fused silica cylindrical resonators is achievable using appropriate manufacturing processes combined with post-processing etching, which offers possibilities for developing high-precision and low-cost cylindrical resonator gyroscopes.

## 1. Introduction

The axisymmetric shell resonator gyroscopes are a type of angular sensor that measures angular rate or angle based on the Coriolis effect. They have triggered great interest because of their comprehensive advantages in measurement accuracy, operation reliability, production cost, etc. [1,2,3]. The hemispherical resonator gyroscope (HRG) and the cylindrical resonator gyroscope (CRG) are typical axisymmetric shell resonator gyroscopes. The HRG has achieved a bias stability of 0.00001 deg/hr, which has been recognized as a disruptive sensor for all applications [4]. Compared with the HRG, the manufacturing of the CRG is simpler and requires a lower cost. For the axisymmetric shell resonator gyroscope, the resonator is the core component, and the quality factor (Q factor) is one of the core parameters limiting the performance of the gyroscope [5,6,7,8,9]. A high Q factor is a prerequisite for a cylindrical resonator gyroscope to reach high precision. In previous reports, the Q factor of the metallic and piezoelectric cylindrical resonators are generally less than 10^5^ due to the materials’ characteristics [10,11,12,13,14,15]. The authors’ group achieved a significant increase in the Q factor by fabricating cylindrical resonators with fused silica [16,17,18].

The Q factors of fused silica resonators are affected by several dissipation mechanisms, including air damping 1*/Q_air_*, anchor loss 1*/Q_anchor_*, thermoelastic dissipation 1*/Q_ted_*, surface loss 1*/Q_surface_*, etc. [19,20,21,22,23]. The total Q can be expressed as [16]:

(1) $$\frac{1}{Q_{total}}=\frac{1}{Q_{air}}+\frac{1}{Q_{anchor}}+\frac{1}{Q_{ted}}+\frac{1}{Q_{surface}}+\frac{1}{Q_{etc.}}$$

However, due to the inherently complicated nature of this dissipation, the overall Q factor is hard to calculate theoretically. The perfectly matched layer is usually used to simulate the anchor loss [20,24], yet there is a significant difference between the simulated and the experimental results. The practical solution to investigating and improving the Q factors of fused silica cylindrical resonators is through the experiment.

Surface loss is recognized as one of the dominating losses, which is far from fully understood. In classical continuum mechanics, the effect of surface energy on the dynamic of resonators is neglected duo to the small ratio of surface area to volume [23]. As the geometry of the resonator is scaled down, the high Q factor is generally offset by increases in energy dissipation from surface effects [25]. Surface loss is considered to be the main reason hindering the miniaturization of high-performance resonators [26]. Nonlocal continuum mechanics provide a new trend for the study of the Q factor improvement of the micro/nanoscale resonator [27]. By comparison, the fused silica cylindrical resonator studied in this paper remained a problem of classical continuum mechanics, and the Q factor can be improved by removing the subsurface damage (SSD). Uchiyama et al. have provided an empirical formula for the surface loss of the cylindrical resonator, which assumes that the surface loss is proportional to the thickness of the SSD layer [28]. Extensive studies have been focused on the analysis and prediction of the SSD for the grinding and polishing of fused silica material [29,30,31,32,33]. Zhong et al. investigated the effect of grinding parameters on the surface/subsurface qualities of fused silica [34]. It is known that the characteristics of SSD change with grinding parameters; however, studies on the features of SSD that affect the mechanical Q factor of fused silica resonators are rarely seen. The relation between the features of the SSD layer and the Q factor of fused silica resonators remains a mystery.

Raman spectroscopies, confocal fluorescent microscopy, and weak absorption can be utilized to characterize the SSD of planar fused silica samples [35,36]. However, these techniques are not readily applicable to fused silica cylindrical resonators due to their complex structure and opaque surfaces. Some studies have focused on the relationship between the surface roughness and Q factor of micro resonators, and the Q factor of micro resonators seem to increase with the decrease in surface roughness [37,38,39,40]. These studies inspired us to monitor the surface roughness of fused silica cylindrical resonators as a parameter for the surface state.

This study intends to experimentally investigate the influences of grinding speed on the Q factor of fused silica cylindrical resonators. Compared with our previous study, in order to further investigate the influence of chemical etching on the Q factor, the etching depth of the resonator is substantially extended. The Q factors and surface roughness of the resonators fabricated with different grinding speeds are measured after chemical etching. Previous useful conclusions are applied in this study for the designing and measuring of fused silica cylindrical resonators. For example, air damping can be significantly reduced by operating the resonator in a low-pressure environment [17]; therefore, the measurement pressure is set as 0.02 Pa to reduce the influence of air damping. The structure of resonators in this study is optimized through a comprehensive consideration of eigenfrequency, thermal elastic damping, and anchor loss [20,21]. We have found, over years of study, that the measured Q factor is significantly affected by the clamping state [41]. Therefore, a torque wrench was used to ensure the same clamping state for different resonators. The measurement results indicate that the Q factors improved significantly during the first few rounds of chemical etching, but they did not improve continuously with the increase in chemical etching depth. Higher grinding speeds result in lower surface roughness and a greater Q factor of the resonator after chemical etching. The significant improvement of Q factor and surface roughness provides favorable conditions for high-quality surface metallized fused silica resonators. During the whole process, the maximum Q factors of the resonator have exceeded 25 million, which is an order of magnitude higher than previous reports, providing an effective reference for the machining and post-processing of fused silica cylindrical resonators with superior Q factors. In addition, this study suggests that the Q factors of fused silica resonators, on the 10^7^ level, are not limited by using a hemispherical or cylindrical structure, and cylindrical resonator gyroscopes can potentially reach the navigation-level performance.

## 2. Experimental Results and Discussion

**Figure 1:**
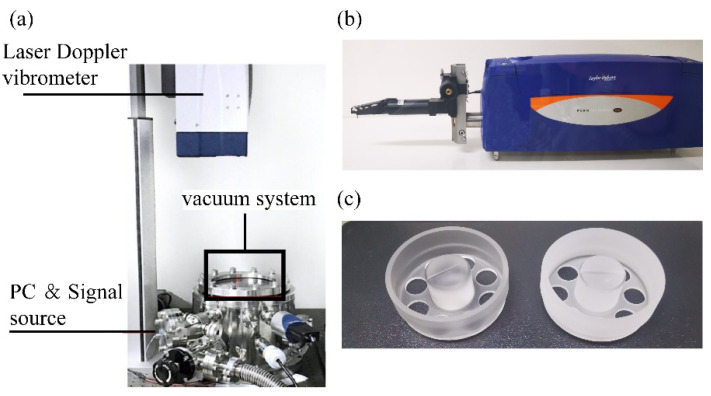
(**a**) The schematic of the measurement system. (**b**) The schematic of the profilometer. (**c**) The schematic of the cylindrical resonator structure.

The structure of the cylindrical resonators, the configuration of the measurement system, and the measurement process are reported in our previous publication [16,18,42]. The measurement system and the cylindrical resonator are shown in Figure 1. Six resonators with the same structure and diameter of 26.4 mm are fabricated with three different grinding speeds, as listed in Table 1. These resonators are chemically etched for thirteen rounds with each round set as 5 minutes. A 20 wt % NH_4_F_2_ solution is used, and the temperature of the solution is kept at 80 °C. The surface roughness, resonant frequencies, and Q factors of the six resonators are measured after each round.

**Figure 2:**
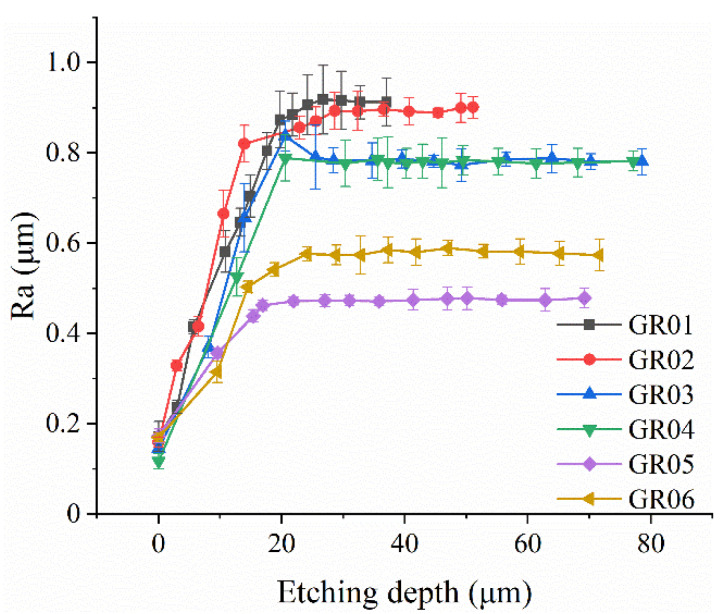
The variation of the surface roughness of six resonators with chemical etching depth.

The surface roughness is measured by a Taylor Hobson profilometer, the assessment length is set to 3 mm, and the measurement position is the outer surface of the resonant shell. Eight different positions on the outer surface of the resonator are measured, and the average value is recorded. It is assumed that the etching depth is consistent over the entire surface of the resonator; the etching depth can be obtained by measuring the mass change of the resonator before and after chemical etching. The variation of the surface roughness with the etching depth is depicted in Figure 2. The relative standard deviation (RSD) of the measured surface roughness is between 1.1% and 11.4%, which might be the result of the uneven distribution of SSD. The measurement results illustrate that the surface roughness of the resonator will gradually reach a stable value after chemical etching, and a higher grinding speed results in lower surface roughness of the resonator eventually. The surface roughness of GR01 to GR06 reached a stable value after 45, 35, 20, 10, 20, and 20 minutes of chemical etching, respectively. The variation of the surface roughness of resonators is less than 3% thereafter. The surface roughness of GR01 to GR06 finally stabilized at 0.91, 0.89, 0.79, 0.78, 0.47, and 0.58 μm, respectively. It is noted that the surface roughness of GR06 is about 0.11 μm larger than that of GR05 after the surface roughness is stable. The surface roughness difference between GR05 and GR06 can be attributed to a degradation in the dynamic balance performance of the grinding tools caused by the excessive grinding speed [34].

**Figure 3:**
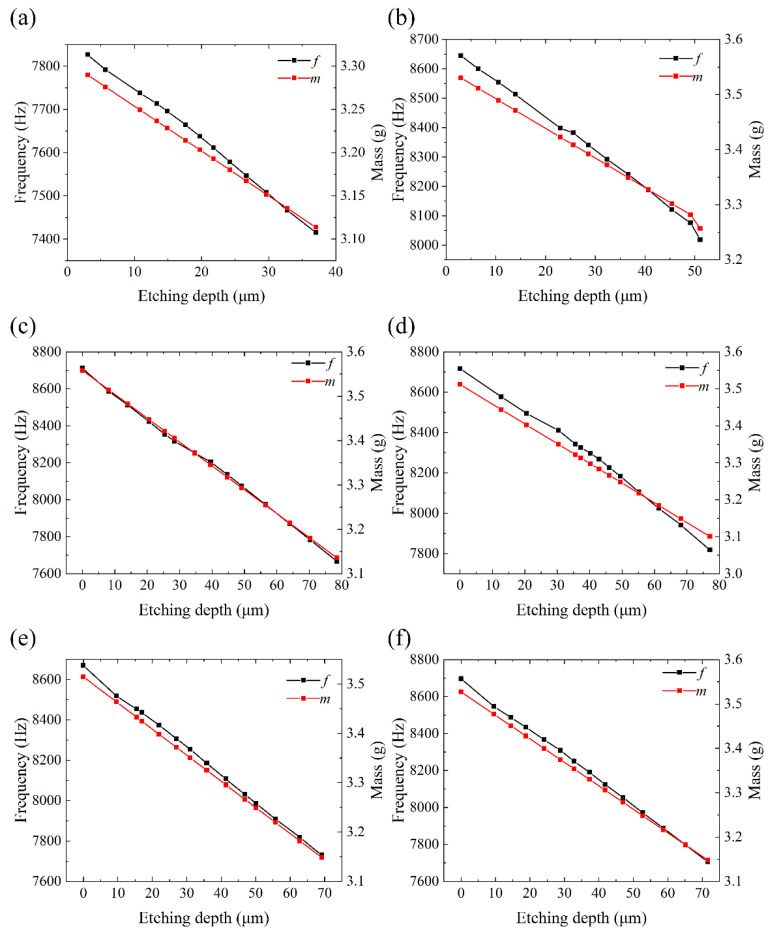
The variation of the resonant frequencies and mass of six resonators with chemical etching depth. (**a**) The variation of resonant frequencies and mass of GR01 with etching depth. (**b**) The variation of resonant frequencies and mass of GR02 with etching depth. (**c**) The variation of resonant frequencies and mass of GR03 with etching depth. (**d**) The variation of resonant frequencies and mass of GR04 with etching depth. (**e**) The variation of resonant frequencies and mass of GR05 with etching depth. (**f**) The variation of resonant frequencies and mass of GR06 with etching depth.

The vibration frequencies of resonators are measured inside the vacuum chamber (0.02 Pa), and the results are depicted in Figure 3. Although the temperature and concentration of the chemical etching solution in each round are strictly controlled, the decreasing of vibration frequencies after chemical etching is not linear. Resonators with different grinding speeds experienced different decreasing patterns of resonant frequencies. Resonators GR01 and GR02 showed an almost linear change of resonant frequencies, while resonators GR05 and GR06 showed an evidently faster change rate during initial etching rounds and a linear slower decreasing rate afterwards. We believe this is due to the fact that the SSD of the resonators fabricated by higher grinding speed is mainly concentrated in the near-surface region. Combined with the measurement results of the surface roughness and the nonlinear theoretical model of the relationship between SSD depth to surface roughness [43], it can be indicated that a larger grinding speed is beneficial to reduce the lateral cracks caused by brittle fracture. It is worth noting that the resonant frequencies and Q factors of the resonator fabricated at the grinding speed of 6.25 m/s cannot be measured under 0.02 Pa before chemical etching, which also indicates that lower grinding speed results in more severe surface damage.

**Figure 4:**
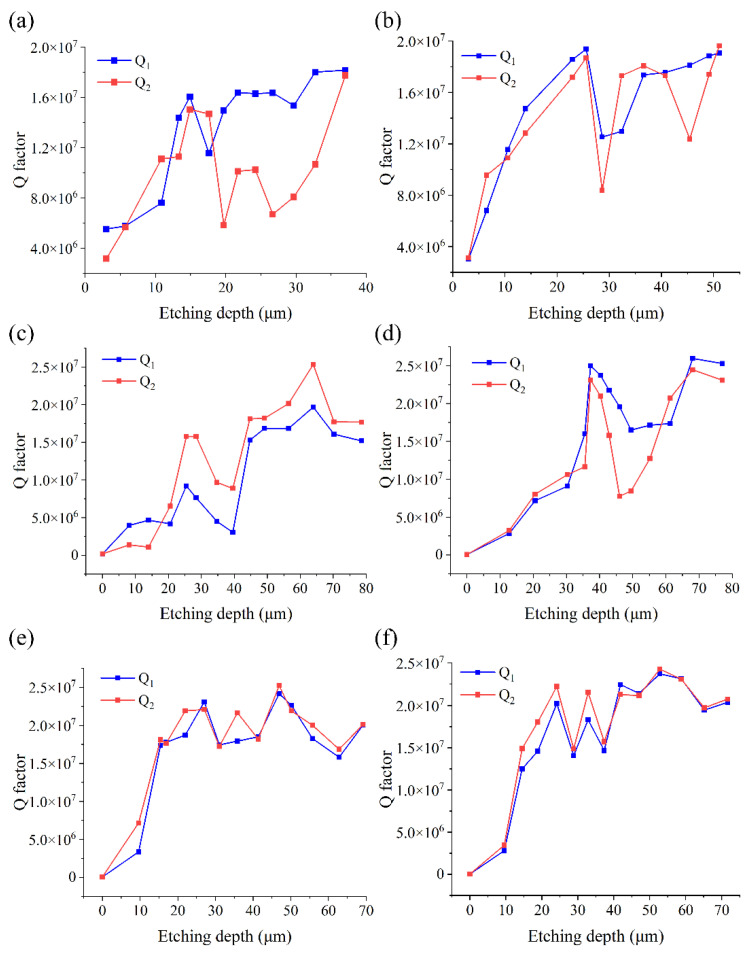
The variation of the Q factors of six resonators with chemical etching depth. (**a**) The variation of Q factor of GR01 with etching depth. (**b**) The variation of Q factor of GR02 with etching depth. (**c**) The variation of Q factor of GR03 with etching depth. (**d**) The variation of Q factor of GR04 with etching depth. (**e**) The variation of Q factor of GR05 with etching depth. (**f**) The variation of Q factor of GR06 with etching depth.

The Q factors of the six resonators are measured inside the vacuum chamber (0.02 Pa), and the results are depicted in Figure 4. It is shown that the Q factors of all six resonators are significantly increased after chemical etching; however, they do not increase continuously with the chemical etching depth. The maximum Q factors of the six resonators appear between 20 and 30 minutes of chemical etching. During the first few rounds of chemical etching, the Q factors increased significantly. Then, they decrease dramatically; however, the Q factors all increased in the subsequent etching, and all six resonators reached over 15 million Q factors. We are yet to understand the mechanism behind the complicated variation pattern of the Q factors with the chemical etching depth. There may be two explanations for the non-continuous increase in the Q factor. Firstly, the uneven distribution of the microcracks in the circumferential direction leads to the uneven circumferential etching, which will cause the uneven mass distribution of the resonator. Uneven mass distribution will result in a significant increase in anchor loss [44]. Secondly, the evolution of the microcracks and scratches of the resonator after grinding could lead to the variation of Q factors through surface loss. However, the mechanism behind this phenomenon is yet to be explained. During the whole process, the Q factor of GR01 to GR06 reached the maximum value of 1.82 × 10^7^, 1.96 × 10^7^, 2.53 × 10^7^, 2.59 × 10^7^, 2.52 × 10^7^, and 2.43 × 10^7^, respectively, after being chemically etched for 65, 65, 55, 60, 45, and 50 minutes. Comparing the maximum Q factors of the resonators fabricated with three different grinding speeds, the Q factors of resonators fabricated with the lowest grinding speed are obviously lower than those of the rest of the resonators. The Q factors of GR05 and GR06 fabricated with the highest grinding speed of 10.41 m/s are slightly different from those of the resonators fabricated at the grinding speed of 8.33 m/s, but their surface roughness is significantly improved. Consequently, an appropriate increase in the grinding speed is beneficial to the improvement of the Q factor of the resonator and the reduction of the surface roughness. Combined with the measurement results of the surface roughness, the experimental results illustrate that the variation of Q factor has no apparent relationship with the surface roughness. Moreover, it is also evident that in contrast with the assumptions of Uchiyama et al. [28], the surface loss is not simply proportional to the thickness of the SSD layer.

## 3. Conclusions

In summary, this paper reports experimental results on the variation of surface roughness and Q factors of fused silica cylindrical resonators with six resonators ground by three different grinding speeds. The results suggest that the Q factors do not have a simple relationship with the depth of the SSD, and the variation of surface roughness has no apparent relationship with the variation of Q factors. The measurement results indicate that a higher grinding speed results in lower final surface roughness and a greater Q factor of resonators after chemical etching. This study also demonstrates that 20 million Q for fused silica cylindrical resonators is achievable using a different grinding process combined with chemical etching, which offers possibilities for developing high-precision and low-cost cylindrical resonator gyroscopes.

## Figures and Tables

**Table 1:** Grinding speeds of resonators.

Resonator Number	GR01	GR02	GR03	GR04	GR05	GR06
Grinding speeds (m/s)	6.25	6.25	8.33	8.33	10.41	10.41
